# Struma ovarii associated with Pseudo-Meig's syndrome and high serum level of CA 125; a case report

**DOI:** 10.1186/1757-2215-5-10

**Published:** 2012-03-21

**Authors:** Nahid Mostaghel, Anahita Enzevaei, Khandan Zare, Masoome Fallahian

**Affiliations:** 1 Department of Obstetrics and Gynecology, Taleghani Hospital, Shahid Beheshti University of Medical Sciences, Tehran, Iran; 2 Infertility and Reproductive Health Research Center, Shahid Beheshti University of Medical Sciences, Tehran, Iran; 3 Department of Pathology, Taleghani Hospital, Shahid Beheshti University of Medical Sciences, Tehran, Iran; 4 Infertility and Reproductive Health Research Center, Shahid Beheshti University of Medical Sciences, Tehran, Iran

**Keywords:** Struma ovarii, Meig's syndrome, CA125

## Abstract

Struma ovarii is a rare form of ovarian neoplasm in a form of mature teratoma and is composed predominantly of thyroid tissue. In the literature review, there has only been 10 cases of this tumor, associated with ascites and pleural effusion (Meig's Syndrome) and increased CA125 so far. In such cases, the tumor mimics malignant ovarian tumor. In this article, the case of a 72-year-old symptomatic woman with a pelvic mass, pleural and peritoneal effusion and high level of serum CA125 is presented. Cytological evaluation for the pleural fluid was performed. She underwent hysterectomy and bilateral salpingo-oophorectomy. The result of pathologic diagnosis is presented in this paper. The patient was well in postoperative period and paraclinical tests including CA 125 were normal as well.

## Background

Struma ovarii is a rare form of ovarian neoplasm derived from germ cells layers in a form of mature teratoma and is composed predominantly of thyroid tissue. The preoperative diagnosis is generally difficult [1]. Although the vast majority of these tumors are benign, they can also mimic malignant ovarian tumors. Elevated tumor markers for a post-menopausal woman presenting with a multilocular adnexal mass, ascites, and pleural effusion can be interpreted as highly suspicious case of malignancy [2]. The diagnosis is usually made postoperatively and by the pathologists. The association of pseudo-Meig's syndrome and elevation of CA125 level to struma ovarii is an extremely rare condition [2] that makes it difficult to differentiate from malignancies [3,4]. In some cases, the tumor produces thyroid hormone and in some cases, hypothyroidism is reported after tumor resection [5].

## Case description

**Figure 1:**
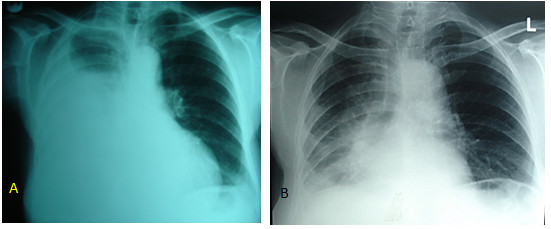
**Patient's chest radiograph before (A) and after (B) operation showing massive pleural effusion that is resolved after surgery**.

**Figure 2:**
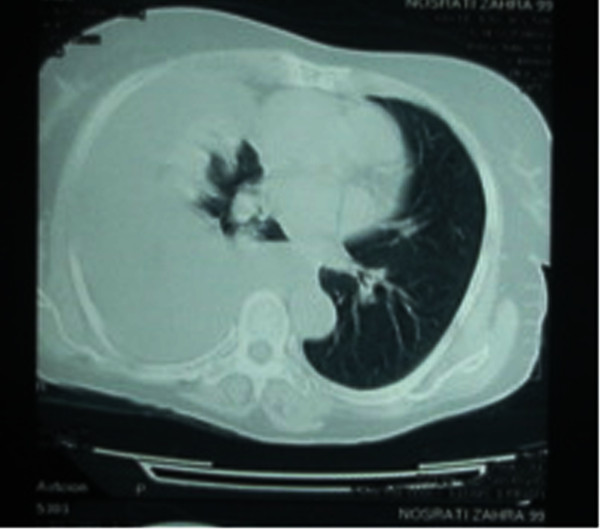
**Chest CT scan of the patient demonstrating pleural effusion**.

A72-year-old woman with a history of gravida 10, para 10 was referred to department of Obstetrics & Gynecology of Taleghani Hospital. She had a pelvic mass that was from 2 months before, and was admitted to a local hospital complaining of dyspnea. Her medical history had included hypertension since 4 years ago and her other medical history had been normal. In physical examination, she had a top normal size uterine and palpable mass in lower side of abdomen. No lymphadenopathy was detected in auxiliary, supraclavicular or inguinal regions. In paraclinical examination, chest imagings showed massive pleural effusion on right side (Figures 1 and 2). The cytological evaluation for the pleural fluid showed inflammatory cells infiltration and no malignant changes had been detected. Then a chest tube was inserted for the patient to reduce the volume of pleural effusion. The pleural fluid culture for anaerobic and other organisms and its direct smear for Mycobacterium Tuberculosis were negative.

Her transvaginal ultrasonography showed multilocular cystic-solid mass on her right ovary measuring 53 mm in diameter, with a 6.8 mm internal septae and 19 mm mural nodule. The low-resistant pattern on the right ovarian artery was in favor of malignancy (RI = 0.61). There was also free intraperitoneal fluid in pelvic cavity. The sonographic figure of her mass declared a case of serous cystadenocarcinoma.

**Figure 3:**
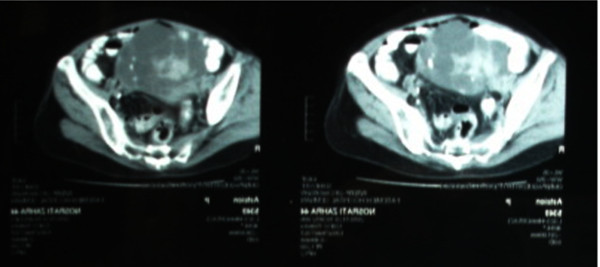
**Pelvic CT scan of the patient showing the mass in the pelvic cavity**.

Then an abdominopelvic CT scan with IV and Oral contrast was performed (Figure 3). The results showed a multicystic pelvic mass with irregular calcified mural nodules, measuring 95*120 mm in size that seemed to be an ovarian cancer. After checking the Tumor markers, the CA125 was higher than normal range (607.4 Units/ml).

CA19-9 = 62.72

CA125 = 607.4

CEA = 1.92

Inhibin A < 0.1

The patient went through operation. Her abdominal cavity had about 700 cc of free fluid sent for cytological examination. The uterine and left ovary was normal. There was a 9 cm-diameter mass on her right ovary with vegetative lesions on its surface that was resected and sent for the frozen section.

**Figure 4:**
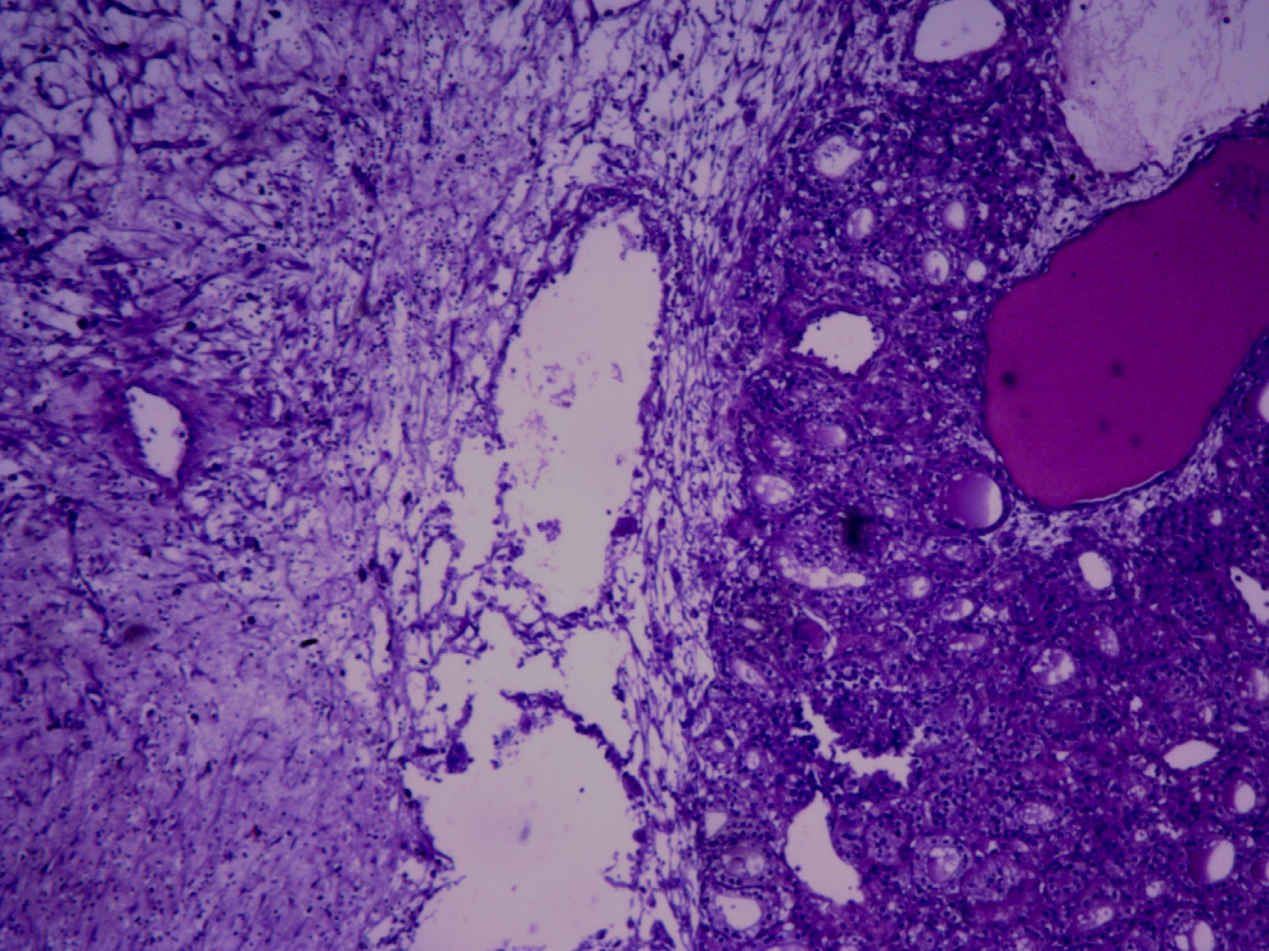
**Short title of figure: Microscopic appearance of the resected tumor**. Detailed legend: The tumor was initially diagnosed as granulosa cell tumor in frozen study but finally turned out to be Struma Ovarii in permanent study.

The result of frozen study was granulosa cell tumor. Therefore, the abdomen was explored and no other involvement was detected. Omentectomy was performed which was intact. The hysterectomy and bilateral salpingo-oophorectomy was performed for the patient. The permanent pathological report was struma ovarii (Figure 4). The uterus, left ovary and fallopian tube were histologically unremarkable and the cytological evaluation of the ascitic fluid showed no evidence of malignant cells.

The patient recovered normally and went to home on the fifth postoperative day. After three months of surgery, no evidence of ascites and pleural effusion was detected and the serum level of CA 125 was in the normal range (9.3 Units/ml); 12 months following the surgery the CA125 level is 12.5 Units/ml, and there is neither pleural effusion in chest X-ray (Figure 1) nor ascites or mass in abdominopelvic ultrasonography. The patient is healthy and TSH level is 1.6 micIU/ml.

## Discussion

Mature cystic teratomas prevalence is about 20% of all ovarian tumors. Thyroid tissue is observed in 5-15% of dermoid tumors, but in struma ovarii tumor the thyroid proportion should be more than 50% of the tissue [5]. Struma ovarii is a mono-dermal variant of ovarian teratoma, which was first described by Von Klden in 1895 and Gottschalk in 1899 [1]. It contains about 2.7% of ovarian teratomas [6]. It is usually a benign lesion but sometimes, malignant transformation could be observed. Preoperative clinical diagnosis of struma ovarii, is very difficult. Despite containing thyroid tissue, only 5% of struma ovarii have features of hyperthyroidism [7]. Ascites has been reported in one-third of cases. However, the association of ascites and hydrothorax with this tumor is not common [1]. Meigs first described the syndrome consisting of ovarian fibroma/thecoma, with ascites and hydrothorax [2]. Pseudo-Meigs syndrome consists of pleural effusion, ascites, and benign tumors of the ovary other than fibromas.

These benign tumors include the tumors of fallopian tube or uterus, mature teratomas, struma ovarii, and ovarian leiomyomas [8]. The ascitic and pleural fluids in Meigs' and pseudo-Meigs' syndrome are usually serous, but may be serosanguinous and can be either transudative or exudative. In electrophoresis performed on several cases it has been determined that pleural and ascitic fluids were similar in nature. Tumor size, rather than the specific histologic type, is thought to be the important factor in the formation of ascites and accompanying pleural effusion. The origin of the effusions remains obscure, although some mechanisms have been suggested such as active fluid secretion by the tumor or peritoneum, venous and/or lymphatic obstruction, low serum protein, inflammatory products, hormonal stimulation, and tumor torsion [8]. In the literature, very few reports have been published on struma ovarii associated to ascites and high CA125 [1,4,9-11].

Other than malignant ovarian epithelial tissue, CA 125 antigen is found on both healthy and malignant cells of mesothelial origin (e.g. peritoneal, pleural, pericardial and endometrial cells) and non-mesothelial cells (e.g. cervical epithelium, amniotic membrane, etc.) [12]. Elevated CA125 is therefore reported in many situations involving these cells, for example ascites or pleural and pericardial effusions. The mechanism of such elevation in CA125 may include infiltration of peritoneum by malignant cells, peritoneal stretching by ascites, low clearance of CA125 by liver in cirrhotic patients, and lymphatic absorption of ascetic fluid full of CA125 antigen. Any condition that can cause ascites or effusions (e.g. cardiac diseases, hepatic failure, infectious processes or malignancies) can raise serum CA125 antigen.

We describe an additional case with struma ovarii associated with pseudo-Meigs syndrome and high level of CA 125. Serum levels of CA-125 can be elevated in Meig's syndrome, but the degree of elevation does not correlate with malignancy [8]. The elevation of CA 125 that may be secondary to the presence of ascites was much higher than that of typically seen with ascites of benign origin.

Association of an ovarian mass with ascites and high CA 125 level in women generally suggests a malignant process. Therefore, the present case with the clinical findings of ascites, hydrothorax, high level of CA 125 and a large pelvic mass in an elderly woman suggested pelvic malignancy before operation. After surgical resection of the tumor and pathologic investigation, the benign nature of the mass became evident and complete remission of the ascites, hydrothorax, and CA125 was obtained after surgery without any further treatment. The patient is on follow-up and now after about 12 months she is symptom free.

Struma ovarii is usually non functional and only 8% of patients present with symptoms and signs of hyperthyroidism, as a result of autonomous activation of the thyroid tissue [13]. At presentation our patient had no findings suggestive of hyperthyroidism and after the operation she is clinically and biochemically euthyroid (TSH = 1.6 micIU/mL). It is worth mentioning that the granulosa-theca cell tumor is probably the most misdiagnosed lesion of the female gonad. According to Emil Novak Ovarian Tumor Registry, lesions misdiagnosed initially in frozen section study as granulosa cell tumors, can be recognized as metastatic carcinomas, teratoid tumors, and poorly differentiated mesothelial tumors [14]. In this case the mass was initially misdiagnosed as granulosa cell tumor but finally the diagnosis of struma ovarii was confirmed.

## Conclusion

According to this report, one should know that there are some benign ovarian masses like Struma Ovarii that resemble malignant conditions initially based on their clinical or laboratory aspects and morphological appearances.

In the differential diagnosis of an ovarian mass in a patient presented with ascites, high CA 125 serum and pleural effusions, but with negative cytologic examination we should consider these benign gynecological conditions too.

## Consent

Written informed consent was obtained from the patient for publication of this Case report and any accompanying images. A copy of the written consent is available for review by the Editor-in-Chief of this journal.

## Abbreviations

CT: Computed tomography; CEA: Carcinoembryonic antigen; CA 125: Carbohydrate antigen.

## Competing interests

The authors declare that they have no competing interests.

## Authors' contributions

AE drafted the manuscript. NM supervised the study. MF and NM are involved in design, interpretation and data preparation. KZ analyzed the patient's tissue samples. All authors had read and approved the final manuscript.

## Authors' information

NM, AE, MF: Department of Obstetrics and Gynecology, Taleghani Hospital, Shahid Beheshti University of Medical Sciences, Tehran, Iran.

KZ: Department of pathology, Taleghani Hospital, Shahid Beheshti University of Medical Sciences, Tehran, Iran.

## References

[B1] Jiang Wei Xin Lu Zhu Zhi L Liu Xi S Xu Cong J Struma ovarii associated with pseudo-Meig's Syndrome and elevated serum CA125: a case report and review of literature J Ovarian Res 2010 3 18 10.1186/1757-2215-3-18 20670426 PMC2923141

[B2] Mitrou S Manek S Kehoe S Cystic struma ovarii presenting as pseudo-Meigs' syndrome with elevated CA125 levels. A case report and review of the literature Int J Gynecol Cancer 2008 18 2 372 375 10.1111/j.1525-1438.2007.00998.x 18334015

[B3] Loizzi V Cormio G Resta L Fattizzi N Vicino M Selvaggi L Pseudo-Meigs syndrome and elevated CA125 associated with struma ovarii Gynecol Oncol 2005 97 1 282 284 10.1016/j.ygyno.2004.12.040 15790478

[B4] Bokhari A Rosenfeld GS Cracchiolo B Heller DS Cystic struma ovarii presenting with ascites and an elevated CA-125 level a case report J Reprod Med 2003 48 1 52 56 12611097

[B5] Willemse PH Oosterhuis JW Aalders JG Piers DA Sleijfer DT Vermey A Doorenbos H Malignant struma ovarii treated by ovariectomy, thyroidectomy, and 131I administration Cancer 1987 60 2 178 182 10.1002/1097-0142(19870715)60:2<178::AID-CNCR2820600210>3.0.CO;2-Q 3297279

[B6] Roth LM Talerman A The enigma of struma ovarii Pathology 2007 39 1 139 146 10.1080/00313020601123979 17365830

[B7] Jing (Jeannie) R Chen, Michel E Rivlin Struma Ovarii 2010 http://emedicine.medscape.com/article/256937-overview

[B8] Klaus-Dieter Lessnau, Carl V Smith Meigs Syndrome 2009 http://emedicine.medscape.com/article/255450-overview

[B9] Long CY Chen YH Chen SC Lee JN Su JH Hsu SC Pseudo-Meigs syndrome and elevated levels of tumor markers associated with benign ovarian tumors-two case reports Kaohsiung J Med Sci 2001 17 11 582 585 11852467

[B10] Mui MP Tam KF Tam FK Ngan HY Coexistence of struma ovarii with marked ascites and elevated CA-125 levels: case report and literature review Arch Gynecol Obstet 2009 279 5 753 757 10.1007/s00404-008-0794-1 18807056

[B11] Jotkowitz MW Gee DC Unique case of massive ascites, extreme elevation of serum CA 125 tumour marker Aust N Z J Obstet Gynaecol 1999 33 4 453 454 8179576

[B12] Silberstien LB Rosenthal AN Coppack SW Noonan K Jacobs IJ Ascites and a raised serum CA 125-confusing combination J R Soc Med 2001 94 581 582 11691896 10.1177/014107680109401107 PMC1282244

[B13] Rana V Srinivas V Bandyopadhyay S Ghosh SK Singh Y Bilateral benign non functional struma ovarii with *Pseudo-Meigs' syndrom* Indian J Pathol Microbiol 2009 52 94 96 10.4103/0377-4929.44978 19136795

[B14] Berek J S Ovarian and Fallopian tube cancer Gynecology 2007 Lippincott Williams & Wilkins, 530 Walnut Street Philadelphia, PA 19106 USA P1521

